# An Information-Theoretic Analysis of Flexible Efficient Cognition for Persistent Sustainable Production

**DOI:** 10.3390/e22040444

**Published:** 2020-04-14

**Authors:** Stephen Fox, Adrian Kotelba

**Affiliations:** VTT Technical Research Centre of Finland, VTT, FI-02044 Espoo, Finland; adrian.kotelba@vtt.fi

**Keywords:** brain entropy, cognitive entropy, efficiency, flexibility, production distributions, relative entropy, sustainability, trade-offs, transfer entropy

## Abstract

Amidst certainty, efficiency can improve sustainability by reducing resource consumption. However, flexibility is needed to be able to survive when uncertainty increases. Apropos, sustainable production cannot persist in the long-term without having both flexibility and efficiency. Referring to cognitive science to inform the development of production systems is well established. However, recent research in cognitive science encompassing flexibility and efficiency in brain functioning have not been considered previously. In particular, research by others that encompasses information (*I*), information entropy (*H*), relative entropy (*D*), transfer entropy (*TE*), and brain entropy. By contrast, in this paper, flexibility and efficiency for persistent sustainable production is analyzed in relation to these information theory applications in cognitive science and is quantified in terms of information. Thus, this paper is consistent with the established practice of referring to cognitive science to inform the development of production systems. However, it is novel in addressing the need to combine flexibility and efficiency for persistent sustainability in terms of cognitive functioning as modelled with information theory.

## 1. Introduction

It has been argued that technological advances will enable expansion of production distributions to more people at more places [1,2]. Also, it has been argued that expanding distributions can improve the sustainability of production [3,4]. Production distributions are an example of coupled human and natural systems [5,6], and as explained within relevant literature, capacity for long-term survival depends on combining flexibility and efficiency. For example, amidst certainty, efficiency can improve sustainability by reducing resource consumption. However, flexibility is needed to be able to survive when uncertainty increases [7,8]. In this paper, flexibility, efficiency, and capacity for long-term survival are analyzed in terms of information (*I*). In doing so, recent research in cognitive science is drawn upon. This research by others encompasses cognitive flexibility and efficiency in terms of brain entropy and applies information-theoretic constructs including information, information entropy (*H*), relative entropy (*D*), and transfer entropy (*TE*) [9,10,11,12,13]. Thus, this paper is consistent with the practice of referring to cognitive science to inform the development of production systems [14,15,16,17,18,19]. However, it is novel in addressing the need to combine flexibility and efficiency for persistent sustainability in terms of cognitive functioning modelled with information theory.

The paper proceeds in five further sections. In Section 2, production distributions are described in terms of information entropy. In Section 3, production distributions are analyzed in terms of relative entropy. In Section 4, flexibility and efficiency are described in terms of cognitive entropy. In Section 5, flexibility and efficiency in production is analyzed in terms of transfer entropy between ecosystems. In Section 6, principal findings are discussed and directions for further research are proposed.

## 2. Information Entropy in Production Distributions

The production of physical goods can be categorized in terms of two fundamental production distributions: flexible production and efficient production. Both can be described in relation to three fundamental trade-offs: product originality, complexity, and unsustainability versus expanding sustainable production distributions. The flexible production distribution is carried out close to the trade-offs. By contrast, the efficient production distribution is carried out away from the trade-offs.

An original product is a product that is designed and produced for the first time. Examples range from bespoke wedding dresses to engineered-to-order superyachts. As expressed in Equation (1), the production of original goods, product-specific information *V* is developed and uncertainty, measured by entropy:

*H*(*U*|*V*) = *H*(*U*) − *I*(*U*;*V*) < = *H*(*U*) (1)

is reduced through iterations of concept sketching, detail design, etc. In this context, *H*(*U*) refers to initial uncertainty about how a product may be manufactured due to the number of possible production options *U*. Eventually, there can be little, if any, *H*(*U|V*) arising from product originality when production is completed and all product details *V* have been recorded fully. The symbol *I*(*U*;*V*) denotes mutual information between production options *U* and product-specific information *V*.

**Figure 1:**
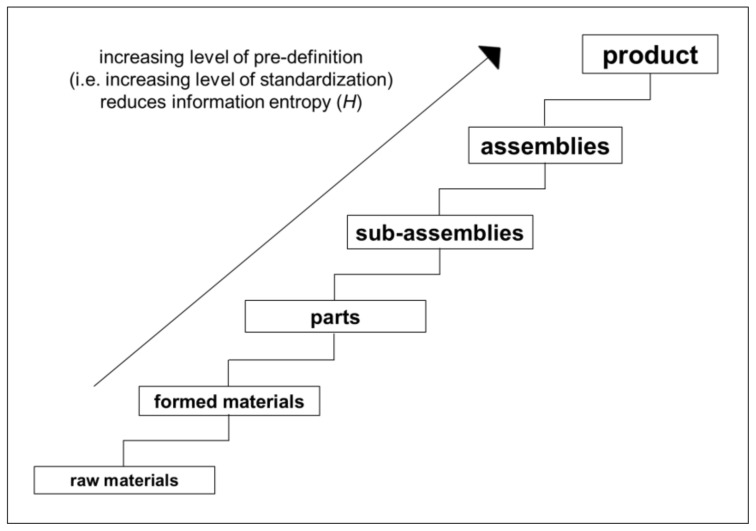
Reducing *H* by moving away from trade-off.

However, product-specific information *V* is of little future use when orders come from amidst the uncertainty of whatever may come to the mind of any individual customer. Resultant production inefficiencies limit potential to expand operations. Hence, in efforts to improve efficiency and expand production, producers seek to move away from the trade-off by reducing product originality [20,21]. As summarized in Figure 1, this moving away from the trade-off involves increasing the pre-definition of product composition up through parts, sub-assemblies, and so on.

The higher the level of pre-definition, the more production efficiency increases and the more production flexibility decreases. This is because production is set-up only to produce only what has been pre-defined. The higher the level of product pre-definition, the lower entropy in production [22], here *H*(*U*).

With regard to the trade-off between product complexity and expanding sustainable production distributions, product complexity is relative. For example, a family car is a more complex product than a bicycle. This is because a family car comprises more part types, more interconnections types, and more interface types than a bicycle. Also, it involves more technologies in provision of more functions. Then, there are exponentially more interactions between parts, interconnections, interfaces, technologies, and functions [23]. Many industrial engineering methods can enable moving away from the trade-off between product complexity and expanding sustainable production distributions [24,25,26]. Reduction of product complexity increases efficiency while reducing flexibility, and entropy [27], here *H*(*U*). In this context, *H*(*U*) refers to uncertainty about how a product may be manufactured due to the number of possible interaction options within its composition. Eventually, there can be little, if any, *H*(*U|V*) arising from product complexity when production is completed and all product details have been recorded.

With regard to the trade-off between product unsustainability and expanding sustainable production distributions, potential for improving production sustainability can be constrained by the unsustainability of the products that are to be made: especially if increasing volumes are to be made [28,29,30]. Some goods can be made of one material that can often be locally sourced, such as furniture made of locally available renewable wood. However, many goods have unsustainable features, such as inclusion of finite natural resources. Furthermore, many goods comprise materials that can seldom be sourced locally, such as rare earths and other scarce materials [31,32]. Thus, there is often long-distance transportation of materials in supply chains characterized by high entropy [33], here *H*(*U*). In this context, *H*(*U*) refers to uncertainty about procurement due to the number of possible procurement options *U*. Eventually, there can be little, if any, *H*(*U|V*) arising from product unsustainability when production is completed and all product details have been recorded. Supply chains can be rationalized to increase their efficiency and reduce *H*(*U*). However, this can lead to supply chains becoming inflexible and prone to failure when situations become more uncertain [34,35].

Although information about one trade-off can be relevant to one or both of the other trade-offs, there is not necessarily causal interaction between the trade-offs. For example, a product that meets an original need does not have to be a complex product nor an unsustainable product. Rather, original products, such as bespoke furniture, can involve few interaction options between parts, technologies, functions, etc., while being made from locally sourced sustainable material. Conversely, an original simple product can be made from a finite material source that is close to extinction. More commonly, many mass produced products are not original and can be increasingly sustainable due to tightening environmental regulations, but are complex with many interdependencies between parts, interconnections, interfaces, technologies, and functions. Hence, zero entropy arising from product originality does not entail zero entropy from complexity and unsustainability. Moreover, zero entropy arising from both product originality and from product unsustainability does not entail zero entropy from product complexity [36].

## 3. Relative Entropy in Production Distributions

The reduction of relative entropy is inherent in both two distributions: flexible production close to the trade-offs and efficient production far from the trade-offs. In particular, reduction of relative entropy between what is expected to happen in production and what actually happens in production. Here, relative entropy can be expressed as *D*(*p||q*) where *p* represents probabilistic prediction of what is expected to happen in production, and *q* represents probabilistic prediction of what should happen in production. *D* can be considered to be concerned with risk management. In particular, minimizing risk by bringing predicted outcomes close to ideal outcomes. Where there is zero relative entropy, there is zero prediction error and zero unwanted surprise from what is produced. For example, if production contributors expect that a production process will conform to specification 99 times in 100, and the predicted process capability is also 99 times per 100, then there will be zero relative entropy [37,38]. Relative entropy is applicable to groups and to individuals. Typically, the flexible production distribution deals with individuals, for example, via bespoke production in exclusive niches. By contrast, the efficient production distribution deals with groups, for example, via mass production for mass markets.

**Figure 2:**
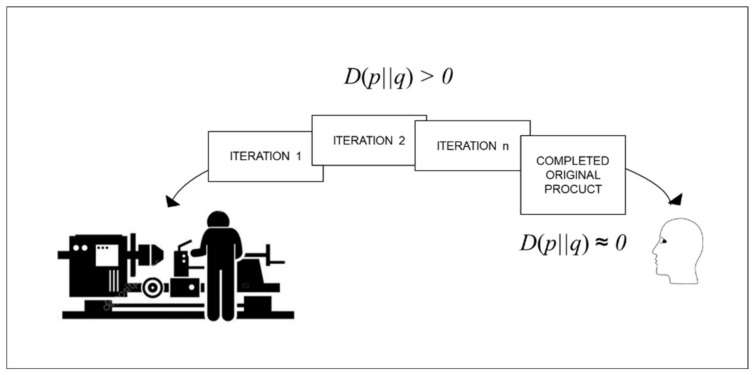
Flexibility to deal with recurring high relative entropy for every order.

As summarized in Figure 2 below, the individual-focused flexible production distribution reduces relative entropy towards zero after customer orders are received. Reduction of relative entropy is carried out through iterations of concept design, detail design, work, and rework, which are needed to transform each customer’s individual idea into a completed original product. The persistence of relative entropy throughout production limits potential for production automation.

In the efficient production distribution, group-focused, concurrent engineering of products and their production processes away from the three trade-offs enables reduction of relative entropy before any orders are received. This is achieved by defining target market segment and specifying typical customer requirements. Then, developing a product that, through market testing, can be predicted to meet typical customer requirements. Concurrently, production processes can be developed to produce the product in accordance with customer requirements. Meanwhile, advertising can be carried out to set customers’ product expectations: i.e., to set what they predict will happen as a consequence of using the product.

**Figure 3:**
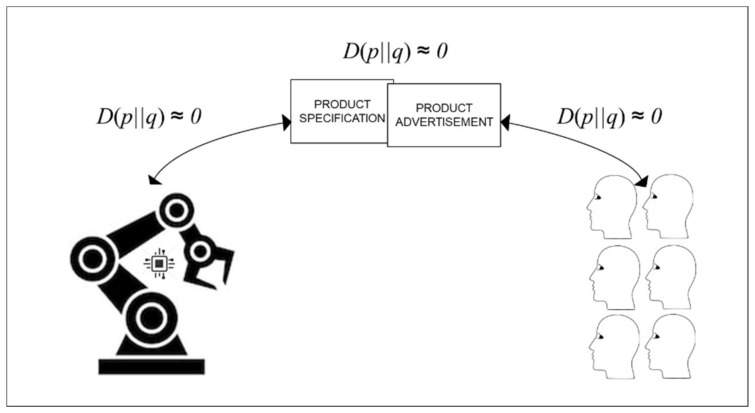
Efficiency from low relative entropy before any product orders are received.

Thus, as summarized in Figure 3 below, there can be zero relative entropy between what customers predict when they order the product and what is produced (i.e., between what is predicted to happen in production and what actually happens in production). The lower the relative entropy before any orders are received, the greater the potential for production automation. In the efficient production distribution summarized in Figure 3 below, some similar practices are used before any orders are received to those used in flexible production distribution shown in Figure 2 above. However, flexible production distribution involves perpetual use of prototyping practices with the additional challenges of what is produced being sold for use in accordance with all relevant regulations for safety and all relevant customer expectations for reliability.

**Figure 4:**
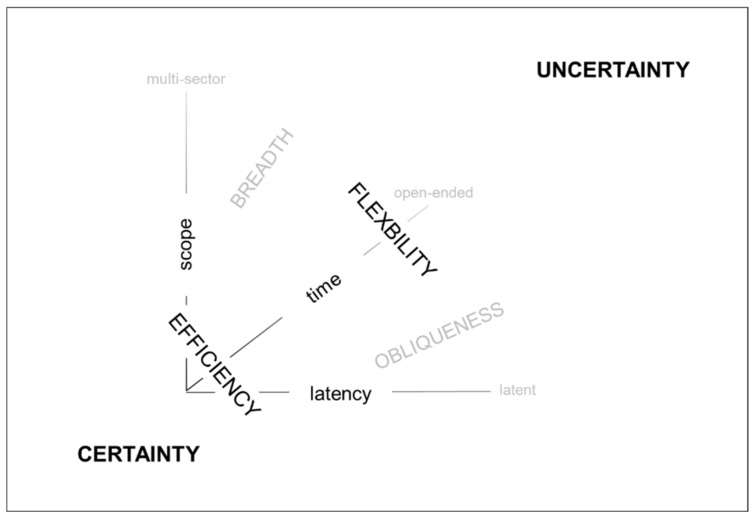
Production distributions related to certainty and uncertainty.

As summarized in Figure 4 below, the flexible production distribution operates where the influence of the trade-offs is broader and more oblique. In particular, the flexible production distribution operates amidst the uncertainty of individual customers’ original ideas, which can have origins in different sectors and can be different every time. Apropos, the constraining influence of the trade-offs can manifest themselves differently in each order that is developed from an individual’s idea into an original product. Thus, in the flexible production distribution, each original product is different to the next—at least at the level of product (Figure 1). Exactly how the constraining influence of the trade-offs will become manifest from one order to the next order is not known in advance. Hence, those who work close to the trade-offs need to be sufficiently flexible to deal with recurring uncertainty. By contrast, as shown in Figure 4, the efficient production distribution operates where the extent of the trade-offs’ influence can be reduced through concurrent engineering during product development.

## 4. Cognitive Entropy for Flexible Efficient Production

Flexibility and efficiency of production contributors can be considered in terms of cognitive entropy (*CE*). This term is derived from brain entropy, which is the number of neural states a given brain can access. The higher the number of neural states that can be accessed the higher the brain entropy [11,12]. The term brain entropy is not compatible with cognitive production that can involve some artificial intelligence as well as natural intelligence [14,15,16,17,18,19]. Rather, cognitive entropy is a more fitting term. Furthermore, rather than consider cognitive entropy as a description of number of neural states that can be accessed, it is more fitting to consider cognitive entropy as a description of number of cognitive models that can be accessed. Model being a term that is compatible with both natural cognition and artificial cognition [39,40]. Thus, *CE* is defined here as the number of cognitive models that a production contributor can access. The higher the number of cognitive models that a production contributor can access, the higher the cognitive entropy. The higher the cognitive entropy, the higher can be flexibility and can be efficiency. In other words, the wider the range of tasks that can be dealt with efficiently [11,12,41].

This is consistent with the construct of adaptive expertise in production work. Adaptive experts can discern the features that differentiate one situation from another. They can understand the significance of those differentiating features. Then, they can modify or invent skills according to the requirements of that situation, and to avoid the unproductive application of previously useful prior learning in new situations [42]. The higher adaptive expertise, the higher can be the extent of work carried out with automaticity: i.e., with minimal cognitive effort [43]. Thus, increasing cognitive entropy (*CE*) among production contributors can reduce *H*(*U*|*V*) in production work by reducing uncertainty about how a task can be carried out right-first-time. Note that a suitable task-specific information *V* is now assumed to be retrieved from a set of cognitive models that a production contributor can access. In this context, suitable task-specific information means information *V* with sufficiently high *I*(*U*;*V*). For example, before increasing *CE* there can be uncertainty about how to carry out a task, which leads to five different task execution options being considered with equal probability i.e., *H*(*U*) = 2.32. Then, increasing *CE* can change the situation to there being certainty about how the task can be carried out right-first-time and hence *H*(*U*|*V*) = 0 due to an information gain *I*(*U*;*V*) of 2.32. The concept of information gain is applied in a variety of fields relevant to production work, including Bayesian experimental design [44], visual neuroscience [45], and robotics [46].

Also, cognitive entropy (*CE*) can lower relative entropy *D(p||q)*. This is because production contributor’s probabilistic prediction of task execution outcome (*p*) is more likely to be the same as the probabilistic prediction of what should happen in task execution (*q*). In particular, when the set of cognitive models denoted by *CE* contains a cognitive model that enables *p* to be equivalent to *q* then relative entropy can be zero. This can be because of at least two reasons. First, production contributors already know what should happen in a wide variety of production situations because they already have a wide variety of relevant cognitive models [11]. Hence, relative entropy *D(p||q)* is immediately lower. Second, high *CE* facilitates flexible original thinking [12], which can facilitate reduction of relative entropy *D* when dealing with a wide variety of production tasks. Conversely, if the set of cognitive models denoted by *CE* does not contain a cognitive model that enables production contributor’s probabilistic prediction of task execution outcome *(p)* to be equivalent to probabilistic prediction of what should happen in task execution (*q)* then relative entropy will not be brought to zero.

From the perspective of physical production work, cognitive models can be made tangible in through a variety of means, including standardized engineering designs or physical kits, which are engineered to be put together right-first-time every time. These tangible means represent the distillation and refinement of the relevant cognitive models of production experts.

**Figure 5:**
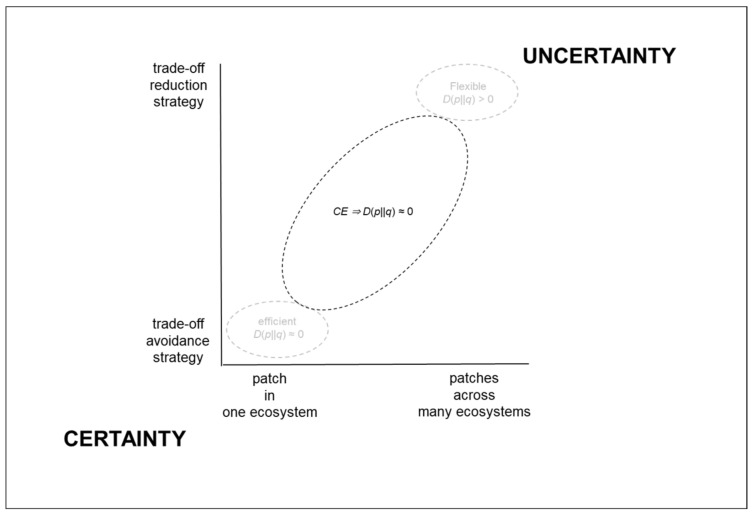
Flexibility and efficiency facilitated by inclusive cognitive entropy.

Cognitive entropy can be enlarged by inclusion of diverse production contributors. This is because groups can have lower prediction error than individuals, and groups with diverse predictive models can have lower prediction error than groups with the same predictive model [47,48]. For example, diversity among production contributors can overcome the tendency for individuals to apply automatically the same cultural perspective to a wide variety of situations: irrespective of the relevance of their cultural perspective to the situation [49,50]. As summarized in Figure 5 below, from an ecological perspective, diversity can be increased by increasing connectivity between patches in different ecosystems [51,52].

## 5. Transfer Entropy for Flexible Efficient Production

**Figure 6:**
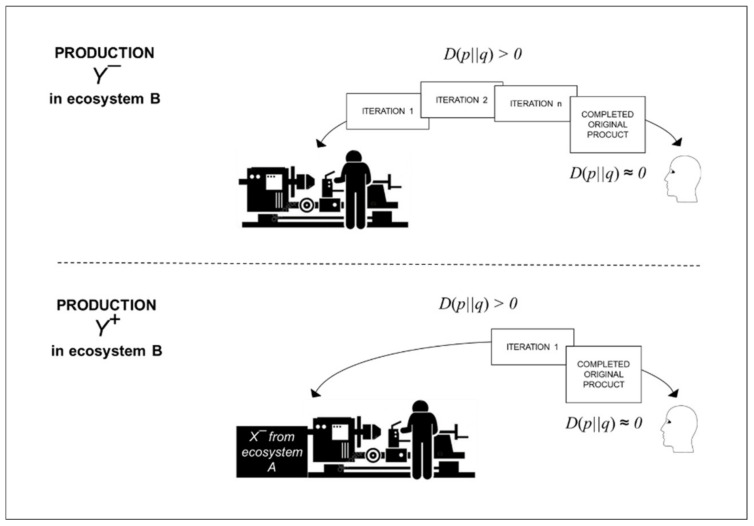
Transfer entropy from one production ecosystem to another.

The amount of information transferred between different ecosystems’ patches can be considered in terms of transfer entropy (*TE*). As expressed in Equation (2):

(2) $$TE\;(X\to Y)=I(X^{-},Y^{+}|Y^{-})$$

where transfer entropy from patch *X* in ecosystem A to patch *Y* in ecosystem B involves additional information contained in past state of patch *X* about future observation of patch *Y* given that the past state of patch *Y* is already known. In summary, the amount of uncertainty in values of $Y^{+}\;$ is reduced by knowing values of *X^−^* given values of *Y^−^* [13]. For example, as summarized in Figure 6, flexible production in ecosystem B can have involved many iterations of concept design, detail design, work, and rework (*Y^−^*). Then, the efficiency of production can be increased in subsequent production ($Y^{+})\;$ by introducing production knowledge into ecosystem B that already exists in ecosystem A. This pre-existing production knowledge from ecosystem A is denoted by *X^−^* in Figure 6.

For example, Open Source Ecology can be described as one of many patches in the open source ecosystem [53]. Open Source Ecology provides a web-based platform for the co-development of production equipment [54]. For capacity building in regions without existing production infrastructure, this patch in the open source ecosystem can be connected to other patches in other not-for-profit ecosystems, such as diaspora networks including engineers [55], which are involved in production in developing countries.

Thus, in this paper, *X^−^* represents pre-existing production knowledge. Pre-existing production knowledge can exist in a wide variety of formats including as engineering designs and as product kits. Both of these examples can be considered to be expressions of cognitive models about production. *Y^−^* represents past production, and $Y^{+}$ represents subsequent production following the introduction of *X^−^*. Here, flexible production is production that encompasses the production of products that have not been pre-defined at product level before customer orders are received (Figure 1). Efficient production is production that can be carried out right-first-time. *TE* is concerned with transfer of information between random processes [56]. Nonetheless, there should be minimum randomness in the production of physical goods. Otherwise, for example, production process will not make right-first-time products that conform to end-user requirements.

Yet, with regard to flexibility, selection of what *X^−^* to combine with *Y^−^* can be unpredictable, and with regard to efficiency, interpretation of *X^−^* by humans and by robots can be unpredictable. This is because, for example, different humans involved in production work have different combinations of physiology, past experiences, personality, gender, culture, which affect differently transitions from sensory inputs to action outputs. In addition, the biases of humans manifest in robots via their human programmers and through human–robot interaction [57,58]. Hence, with regard to flexibility, labelling of *X^−^* needs to be related to those *Y* to which it is relevant. In doing so, mental models in ecosystems A (source of *X^−^*) and B (source of *Y*) need to be taken in account [59]. With regard to efficiency, side information provided by *X^−^*, needs to reconcile probabilistic prediction of task execution outcome (*p*) with what should happen in task execution (*q*) and so reduce relative entropy *D*(*p*||*q*) towards zero.

## 6. Integrated Analysis of Flexible Efficient Production

From the perspective of increasing capacity for persistent sustainable production, increasing *CE* involves expanding access to cognitive models, which can provide information that can enable a wider range of tasks (i.e., flexible) to be executed right-first-time (i.e., efficiency*)*. We conjecture that higher *CE* enables production flexibility that can lead to a wider breadth of production efficiency [11,12,41]. Cognitive models from *CE* can provide information that reduces relative entropy *D*(*p*||*q*) for $Y^{+}$ when *X^−^* is introduced to better enable $Y^{+}$. In particular, production contributor’s probabilistic prediction of task execution outcome (*p*) in $Y^{+}$ is more likely to be the same as the probabilistic prediction of what should happen (*q*) in task execution $Y^{+}$.

**Figure 7:**
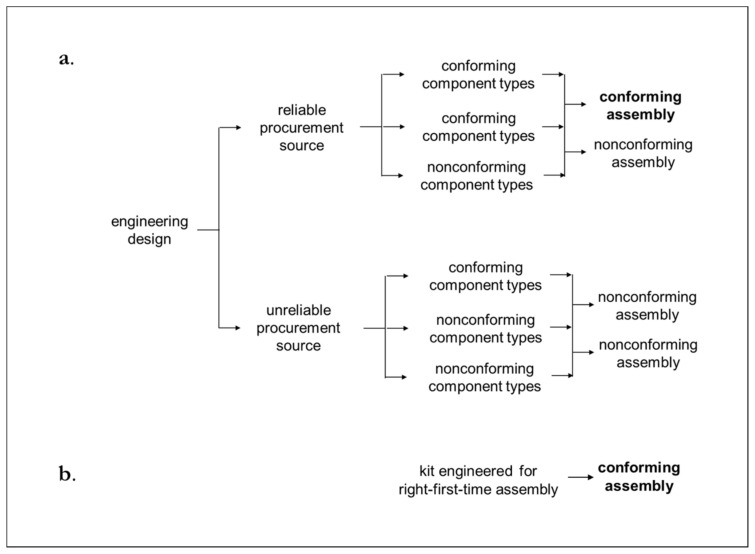
Higher potential for *H* in (**a**) engineering design than in (**b**) engineered kit.

Achieving this ideal may not be possible in practice. However, there are many opportunities to substantially reduce entropy *H*(*U*|*V*) and *D*(*p*||*q*) through well-established industrial engineering practices. For example, production *H*(*U*|*V*) and *D*(*p*||*q*) based on engineering designs (Figure 7a) can be improved upon if *X*^−^ is engineered kits (Figure 7b). This is because with engineering designs there can be different interpretations of component details, from where to procure components, and how components should be assembled. By contrast, kits are a well-established method for reducing *H*(*U*|*V*) and aligning production contributor prediction of task execution outcome (*p*) with what should happen in task execution (*q*). As summarized in Figure 7b, this is because all of a kit’s components are designed and provided for ease of assembly to the extent that there can be only one way to assemble one component with another [60]. An example where *CE* reduces *H*(*U*|*V*) and *D*(*p*||*q*), but not to zero, is described in the calculations and two tables below. The example involves manufacturing and installation and includes engineering design and engineered kit.

In particular, *Y^−^* manufacturing actions based on engineering design (Figure 7a) can be actioned in four different ways with equal probability, which leads to *H*(*U_m_*) = 2.00. Installation work can involve higher uncertainty than manufacturing because installation settings vary from order to order. Hence, *H*(*U_i_*) = 2.32 when there are typically five different ways with equal probability of carrying out the installation work. Thus:

*H*(*Y^−^)* = H(*U_m_, U_i_*) = *H*(*U_m_*) + *H*(*U_i_*) = (2.00 + 2.32) = 4.32 (3)

because manufacturing and installation processes are considered to be independent. Table 1 provides a summary of relative entropy *D*(*Y^−^*) where manufacturing actions are based on engineering design. As expressed in Equation (4), relative entropy *D_m_*(*p*||*q*) for the manufacturing aspects of *Y^−^* is 4.17. This is because manufacturing can be expected (*p*) to be carried out right-first-time once in every four attempts {0.25,0.75}, but it should be carried out (*q*) at least 99 times in every 100 attempts {0.99,0.01}.

*D_m_(p||q) = D*({0.25,0.75}||{0.99, 0.01}) = 0.25*log_2_(0.25/0.99) + 0.75*log_2_(0.75/0.01) = 4.17 (4)

As expressed in Equation (5), the higher uncertainty of installation work in *Y* leads to *D* = 4.59 where *p* = {0.20,0.80} and *q =* {0.99,0.01}. This is due to the production contributor’s probabilistic prediction of task execution outcome (*p*) is correct execution of installation work in *Y* once in every five attempts, but what should happen (*q*) is correct execution of installation work at least 99 times in every 100 attempts.

*D_i_ (p||q) = D*({0.20, 0.80}||{0.99, 0.01}) = 0.20*log_2_(0.20/0.99) + 0.80*log_2_(0.80/0.01) = 4.59 (5)

As shown in Table 1, total relative entropy *D* is 8.76. This is because *p* in manufacturing and *p* in installation are independent distributions, and *q* likewise. For example, manufacturing is done in a factory setting, but installation is done in a much less controlled setting.

**Figure 8:**
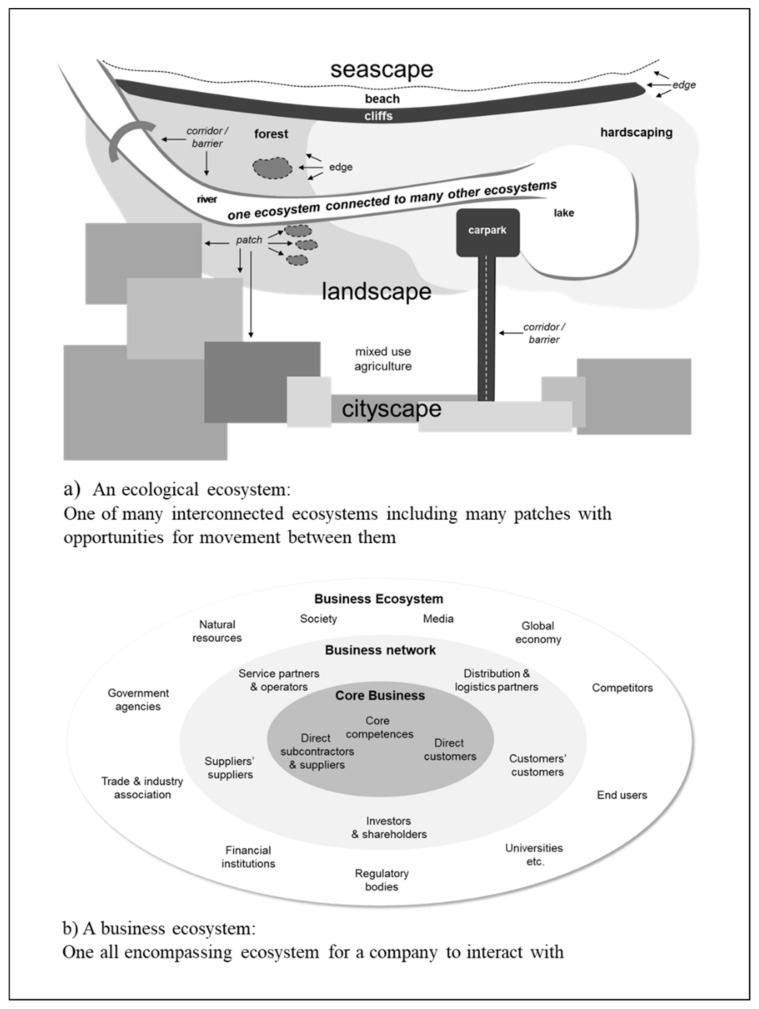
Comparison of representations of ecological ecosystem (**a**) and business ecosystem (**b**).

An engineered kit can be made only one way (Figure 8b): i.e., *H*(*U_m_*) = 0. Consider an everyday example of kitchen cupboard kits. These can have standardized height and modular widths, e.g., 30, 60, 90, and 120 cm. These flat-pack kits obviate the need to make kitchen cupboards uniquely for each kitchen based on kitchen-specific measurements. Unlike the production of unique cupboards for kitchen-specific measurements, there can be designated task-specific production equipment that can be set-up to enable kits to be manufactured right-first-time. Thus, a manufacturer in ecosystem B does not have to import kits produced in ecosystem A. Rather, a manufacturer in ecosystem B can transfer the relevant cognitive model from ecosystem A that enables the right-first-time manufacture of modular kitchen cupboard flat-pack kits. Also, the introduction of modular kits can reduce uncertainty in manufacturing. This is achieved by having end panels that are designed to be rapidly fixed right-first-time to assembled cupboards that are close to kitchen walls. The remaining uncertainties are what widths to cut end panels to abut against walls that may not be entirely vertical and straight. This can lead to the number of ways to carry out installation being reduced from five to two, and as a consequence *H(U_i_)* being reduced from 2.32 to 1.00. Thus, *H(*$Y^{+}$*)* = *H*(*U_m_*) + *H*(*U_i_*) = (0.00 +1.00) = 1.00. Hence, when *X^−^* is in the form of an engineered kit, information gain:

(6) $$I=H(Y^{-})-H(Y^{+})=4.32-1.00=3.32$$

In other words, replacing engineering design with engineered kit leads the amount of uncertainty in values of $Y^{+}\;$ being reduced by 3.32 from 4.32 to 1.00.

A summary for relative entropy *D(*$Y^{+}$*)* with kit (Figure 7b) is provided in Table 2. As expressed in Equation (7), relative entropy *D*(*p||q*) for manufacturing work in $Y^{+}$ is 0.00 because (*p*) is the same as (*q*). That is the probabilistic production of task outcome (*p*) is that work will be carried out right-first-time at least 99 times in every 100 attempts, and work should (q) be carried out right-first-time at least 99 times in every 100 attempts.

*D_m_(p||q) = D*({0.99,0.01}||{0.99, 0.01}) = 0.99*log_2_(0.99/0.99) + 0.01*log_2_(0.01/0.01) = 0.000 (7)

As expressed in Equation (8), relative entropy *D*_i_(*p||q*) for installation work in $Y^{+}$ is 2.39.

*D_i_(p||q) = D*({0.50,0.50}||{0.99, 0.01}) = 0.50*log_2_(0.50/0.99) + 0.50*log_2_(0.50/0.01) = 2.33 (8)

Thus, there is a reduction in relative entropy from *D(Y^−^)* = 8.76 to *D(*$Y^{+}$) = 2.33.

## 7. Discussion

### 7.1. Principal Findings

This paper is consistent with the established practice of referring to cognitive science to inform the development of production systems [14,15,16,17,18,19]. However, it is novel in addressing the need to combine flexibility and efficiency for persistent sustainability in terms of cognitive functioning as modelled with information theory. In particular, the study reported in this paper has been informed by research by others that encompasses cognitive flexibility and efficiency in terms of brain entropy and applies information-theoretic constructs including information (*I*), information entropy (*H*), relative entropy (*D*), and transfer entropy (*TE*) [9,10,11,12,13]. As summarized in Table 3, application of these information-theoretic constructs has revealed underlying issues among interrelationships between flexibility and efficiency in physical production work.

### 7.2. Directions for Future Research

Previous production research has applied information theory [22,27,33] and considered some aspects of ecology, in particular ecosystems [61]. However, there has been little consideration of findings from ecological studies that there needs to be flexible efficiency long-term sustainability. For example, as summarized in Figure 8a, fundamental ecological constructs are not considered, such as patches and connectivity between them [51,52], in previous research concerned with applying information theory to sustainability of production distributions.

Rather, as summarized in Figure 8b, ecosystem is applied in management studies, including those related to production, as a broad metaphor [62]. Accordingly, future research could investigate the applicability of diverse information-theoretic ecological studies concerned with combining flexibility and efficiency for long-term sustainability [63,64,65]. Particularly relevant can be ecological studies concerned with issues related to cognition such as the transfer of memory from one ecosystem to another. The ecological memory of an ecosystem can be defined as its information legacies from past dynamics [66] and various means of transfer include entities that can move relevant ecological memory between ecosystems [67].

## Figures and Tables

**Table 1:** *D(Y^−^)* Summary.

*Y^−^*		
*D* Manufacture	*D* Installation	*D* Manufacture and Installation
4.17	4.59	8.76

**Table 2:** *D*($Y^{+}$) Summary.

$Y^{+}$		
*D* Manufacture	*D* Installation	*D* Manufacture and Installation
0.00	2.33	2.33

**Table 3:** Principal findings.

Type of Entropy	Production Issue		
	Flexibility	Efficiency	Flexible Efficiency
Information entropy (*H*)	Can deal with high *H* but not efficiently	Depends upon low *H*	Restricted by need to deal with high *H*
Relative entropy (*D*)	*D* is high when every order is received	*D* reduced before any orders are received	Restricted by need for low *D*
Cognitive entropy (*CE*)	High *CE* compatible with flexibility	High *CE* is compatible with efficiency	Depends upon access to *CE*
Transfer entropy (*TE*)	*TE(X→Y)* can increase flexibility when *X* is outside of extant representations of cognitive models in Y	*TE(X→Y)* can increase efficiency when *X* has lower *H* than extant representations of cognitive models in Y	*TE(X→Y)* can reduce *H* and *D*
